# Exploring the relationship between social exclusion and smartphone addiction: The mediating roles of loneliness and self-control

**DOI:** 10.3389/fpsyg.2022.945631

**Published:** 2022-08-15

**Authors:** Heng Yue, Xiwen Yue, Xuemin Zhang, Bo Liu, Hugejiletu Bao

**Affiliations:** ^1^School of Psychology, Inner Mongolia Normal University, Hohhot, China; ^2^Beidou College, Wuhan Qingchuan University, Wuhan, China; ^3^College of Physical Education, Inner Mongolia Normal University, Hohhot, China

**Keywords:** social exclusion, smartphone addiction, loneliness, self-control, mediating effect

## Abstract

Previous studies have identified many antecedents of smartphone addiction. However, social exclusion as a risk factor for smartphone addiction has not been widely studied, and little is known concerning the psychological mechanism underlying this association. The present study tested the influence of social exclusion on smartphone addiction as well as the mediating roles of loneliness and self-control in this relationship. An online survey was conducted, and the sample consisted of 573 university students (323 females). The results revealed that (1) social exclusion was a positive predictor of smartphone addiction; (2) loneliness and self-control separately mediated the association between social exclusion and smartphone addiction; and (3) loneliness and self-control sequentially mediated the relation between social exclusion and smartphone addiction. Possible explanations were discussed. The findings of the current study would contribute to understanding the relationships between these study variables as well as the psychological mechanisms underlying these associations.

## Introduction

Smartphones have significantly changed our daily lives. Since millions of applications have been developed and installed on this internet-enabled intelligent device, nowadays, people are capable of communicating with others, listening to music, watching videos, as well as playing games. Some studies have indicated that smartphone usage can expand horizons, provide convenience, promote safety, alleviate pressure, and facilitate learning (Elhai et al., 2017; Hong et al., 2019). Therefore, the smartphone has become a “versatile baby-sitter” and has penetrated into nearly every aspect of our lives (Shen and Wang, 2019). Due to the fact that smartphones have brought great convenience to our daily lives and they can be used almost anytime and anywhere, an increasing number of people tend to spend plenty of their time on them. In this way, they may develop immoderate usage habits (such as overuse and abuse), which can bring about many detrimental consequences. Smartphone addiction is one of the maladaptive usage behaviors (Xie et al., 2019), and it has gained a lot of attention from social scientists and the general public.

Smartphone addiction is defined conceptually as excessive smartphone use with associated functional impairment and resulting symptoms similar to those seen in other addictive behaviors such as withdrawal and tolerance (Billieux et al., 2015; Elhai et al., 2019). Previous studies have found that smartphone addiction can lead to a series of mental health problems, such as depression, loneliness, anxiety, stress, low self-esteem, low self-efficacy, and so on (Lee, 2017; Lapierre et al., 2019; Wang et al., 2019; Kara et al., 2021). Some scholars have verified that problematic smartphone usage can contribute to poor sleep quality and other physical symptoms such as eye syndromes, body fatigue, physiological dysfunction, and weakened immunity (Berolo et al., 2011; Choi et al., 2018; Xie et al., 2018; Yang et al., 2019). Besides, researchers have found that smartphone addiction can bring about academic procrastination, which may have adverse effects on addicts’ academic performance (Ji et al., 2014; Hawi and Samaha, 2016). Since smartphone addiction can induce so many negative influences, it is imperative for researchers to investigate the antecedents and the underlying psychological mechanisms that may put individuals at the risk of this addictive behavior. This will not only be conducive to understanding the associations between these variables, but also contribute to the effective prevention and early intervention of this behavioral disorder.

Plenty of previous studies have provided empirical evidence for the relationships between the antecedents (such as depression, anxiety, stress, loneliness, personality) and smartphone addiction. However, one important antecedent—social exclusion—has not been widely studied. The psychological mechanisms underlying the relationship between social exclusion and smartphone addiction are still unclear. To this end, by constructing a sequential mediation model, the present study aimed to examine the relationship between social exclusion and smartphone addiction as well as investigating the mediating roles of loneliness and self-control.

### The relationship between social exclusion and smartphone addiction

Social exclusion is defined as the sensation of being physically (e.g., social isolation) or emotionally isolated from others (Wesselmann et al., 2016). Social exclusion involves two core experiences rejection and ostracism. Rejection is defined as being explicitly or implicitly told that one is unwelcome in a social relationship; ostracism is commonly characterized by being overlooked and excluded by a person or group (Wesselmann et al., 2016). One previous study has indicated that individuals who experience a sense of social exclusion tend to have heightened requirements for attention. This will result in their attaching to social media (David and Roberts, 2017). Besides, researchers have found that social exclusion may contribute to Facebook addiction (Lim, 2019). On the one hand, this could be because people are afraid of being excluded and forgotten, which motivates them to spend more time and energy on Facebook; on the other hand, researchers have confirmed that social exclusion can cause social anxiety and motivate people to restore their social affiliation, which can lead to addictive behavior (Lim, 2019). In addition, some scholars have verified that social exclusion can significantly predict increased internet addiction. In their opinion, being excluded by other individuals leads people to experience enhanced negative emotions, decreased self-regulation, as well as impaired self-control, which may contribute to internet addiction (Poon, 2018; Arslan and Coşkun, 2021). Based on this, the present study hypothesizes (H1): social exclusion can significantly and positively predict the severity level of smartphone addiction.

### The mediating role of loneliness

Loneliness has been proved to be an import consequence of social exclusion. Loneliness refers to the negative emotional reaction to the difference between one’s anticipated and actual social relationship (Peplau and Perlman, 1982; Vanhalst et al., 2015). Previous research has demonstrated that some indicators of social exclusion (such as lower frequency of social contacts and lack of emotional support) can significantly and positively predict loneliness experiences (Dahlberg et al., 2022). Prior scholars also proved that social exclusion has a strong impact on students’ loneliness as well as other mental health problems (Arslan, 2021). Besides, one empirical research confirmed that socially excluded individuals report more loneliness experiences than those who were not excluded (Kavakli, 2019). Moreover, other studies also provide evidence for the association between social exclusion and loneliness (Leary, 1990; Li et al., 2019; Arslan and Yıldırım, 2021). According to the need-to-belong theory and the temporal need-threat model (Williams, 2009; Baumeister, 2011), social exclusion can lead to a lack of belongingness, which can exacerbate feelings of loneliness. Therefore, loneliness as a result of social exclusion can be comprehended.

Loneliness has been proved to be correlated with smartphone addiction by plenty of studies. Researchers indicated that individuals with loneliness experiences are more prone to overusing cyber-technological devices (Enez Darcin et al., 2016). Empirical research showed that loneliness can positively and significantly predict the pattern of smartphone usage and the severity of smartphone addiction (Bian and Leung, 2015). According to the compensatory internet-use model, people often use smartphones to alleviate negative emotions or compensate for psychosocial problems (Kardefelt-Winther, 2014). Therefore, lonely individuals may turn to smartphones to relieve their dysphoric moods or seek compensation for their psychological requirements. Ultimately, frequent, intensive, and excessive use will lead them to smartphone addiction.

From what has been mentioned above, the present study hypothesizes (H2): loneliness mediates the relationship between social exclusion and smartphone addiction.

### The mediating role of self-control

The negative impact of social exclusion on self-control has been verified by numerous prior studies. Previous scholars have found that social exclusion is negatively and significantly associated with self-control (Crescioni and Baumeister, 2009; Burson et al., 2012; Xiaojun et al., 2017). Some scholars have also indicated that socially excluded individuals are more likely to behave aggressively than those who are not excluded (Ren et al., 2018). In addition, social exclusion can impair self-regulation (Stenseng et al., 2015), hinder the inhibitory capacities (Sato et al., 2018), lead to impulsive behaviors (Luo et al., 2021) as well as plenty of negative affects (such as loneliness; Arslan, 2021), which will decrease individuals’ self-control (Chester et al., 2016). Moreover, because one of the primary goals of self-control (or self-regulation) is to get approval from others (Baumeister et al., 2005), according to the need-to-belong theory and the temporal need-threat model (Williams, 2009; Baumeister, 2011), individuals’ self-control capacity will be impaired when they are socially excluded. Therefore, self-control could be considered as one of the detrimental consequences of social exclusion.

Low self-control has been confirmed as one of the major antecedents of smartphone addiction. Previous studies verified that self-control is a protective factor against individuals’ smartphone addiction; the self-control score of the smartphone addiction risk group is significantly higher than that of the general group (Kim et al., 2018; Sok et al., 2019). Empirical studies have also revealed that ego-depleted individuals (with low self-control) exhibit more reward-sensitivity than the non-depleted participants (Giacomantonio et al., 2014). One behavioral and electrophysiological study showed that low self-control could lead to impulsive behavior (Dou et al., 2014). All of these consequences have been proved to be important predictors of smartphone addiction (Kim et al., 2016; Deng et al., 2021). According to the strength model of self-control (Baumeister et al., 2007), self-control relies on limited energy resource. Inadequate self-control will not only lead to impulse-control problems such as alcohol and drug abuse as well as smartphone addiction, but also bring about emotional problems that can further contribute to smartphone addiction. Therefore, it is reasonable to hypothesize that low self-control is a predictor of smartphone addiction.

Based on the results of theoretical and empirical studies, the present study hypothesizes (H3): self-control has a mediating role between social exclusion and smartphone addiction.

### The sequential mediation model

The relationship between loneliness and self-control has been well documented by empirical studies (Liu et al., 2017; Li et al., 2021). Some scholars indicated that loneliness can promote internet addiction and Facebook addiction through low self-control (Özdemir et al., 2014; Iranmanesh et al., 2021). According to the Interactions of Person-Affect-Cognition-Execution (I-PACE) model (Brand et al., 2016), specific internet-use disorders result from the interactions between social cognition factors (such as perceived social exclusion) as well as the mediators -- affective and cognitive responses (such as loneliness and reduced self-control). Therefore, the present study put forward the following hypothesis (H4): loneliness and self-control can sequentially mediate the association between social exclusion and smartphone addiction.

### The present study

**Figure 1:**
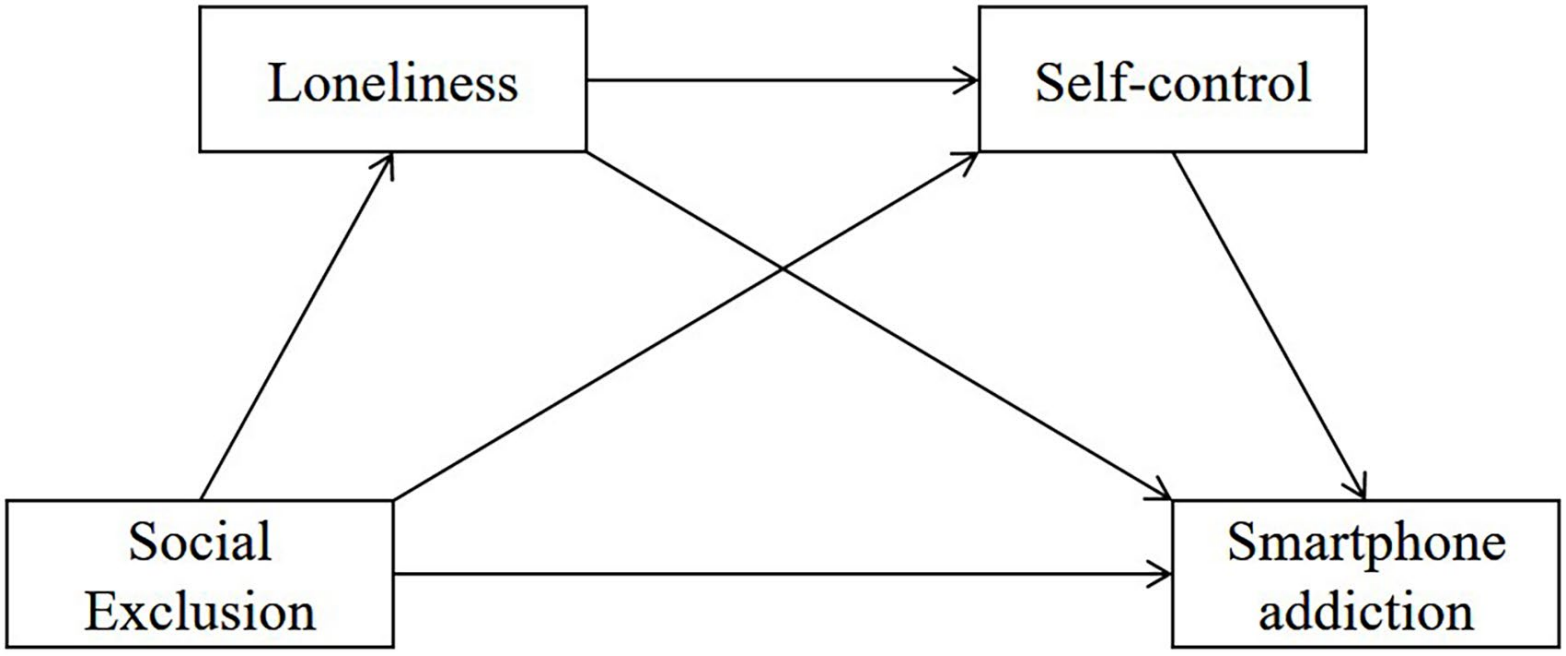
The sequential mediation model.

In the current study, a sequential mediated model (Figure 1) was constructed to examine the relationship between social exclusion and smartphone addiction as well as the possible mediating mechanisms. The hypotheses of the present study were that (1) social exclusion would be positively correlated with smartphone addiction; (2) loneliness would mediate the association between social exclusion and smartphone addiction; (3) self-control would mediate the association between social exclusion and smartphone addiction; and (4) loneliness and self-control would be two sequential mediating mechanisms in the relationship between social exclusion and smartphone addiction.

## Materials and methods

### Participants and procedure

An online survey was designed and performed to collect information concerning the study variables. The final sample comprised 573 university students, and there were 250 males and 323 females. The average age of the total participants was 20.20 ± 1.61 years old, ranging from 18 to 25 years of age.

This study was approved by the ethical committee of the authors’ institution. Participants were recruited from five universities in Inner Mongolia, China. All the participants should be university students, and they should be at least 18 years old. They should have a smartphone, and they should be able to use the phones to complete the questionnaires. The survey was conducted during the spare time of the students, such as breaks between classes and after class time. Before sending out the link to the questionnaire, all the participants were verbally told that this survey was anonymous, they were not forced to complete the questionnaires, their data would be used merely for scientific research, and would be kept confidential. They could stop their participation anytime they wanted to, and this would not have any bad effects on them. This content was also presented as instructions in front of the formal questionnaire. After obtaining the verbal informed consent from the participants and their teachers, the link to the questionnaire was shared in the students’ WeChat or QQ groups. They could complete these measurements without any restrictions.

### Measurements

#### Social exclusion

Social exclusion was assessed by the Ostracism Experiences Scale (Carter-Sowell, 2010). This scale contained 8 items (e.g., “In general, others leave me out of their group”). Participants were asked to rate each item on a seven-point scale, ranging from 1 (hardly ever) to 7 (almost always). Higher sum scores indicated more ostracism experiences. In the present study, the internal consistency of this scale was high (Cronbach’s α = 0.831).

#### Loneliness

Loneliness was measured by the short-form UCLA Loneliness Scale (ULS-6; Xu et al., 2018). This instrument had 6 items (e.g., “I lack companionship”), all items were answered on a four-point Likert scale (from 1 “never” to 4 “always”), with higher scores representing greater degrees of loneliness. In this study, the internal consistency of this scale was high (Cronbach’s α = 0.861).

#### Self-control

Self-control was assessed by the Brief Self-Control Scale (Morean et al., 2014). This scale comprised seven items (e.g., “I am good at resisting temptation”). Items were scored on a five-point Likert scale ranging from 1 “not at all” to 5 “very much” (except four items that were reverse scored). Higher sum scores on this scale indicated higher self-control capacity. In the present study, the internal consistency of this was good (Cronbach’s α = 0.735).

#### Smartphone addiction

Smartphone addiction was measured by the Smartphone Addiction Scale (Kwon et al., 2013). This scale contained 10 items (e.g., “Missing planned work due to smartphone use”). Participants were asked to rate their levels of smartphone addiction on a six-point Likert scale (1 = “strongly disagree”; 6 = “strongly agree”). In the present study, the result of the internal consistency of this scale was good (Cronbach’s α = 0.876).

### Statistical analysis

SPSS 25.0 software was applied to perform descriptive statistics, correlation, reliability, and linear regression analysis. SPSS PROCESS macro program (model 4) was employed to test the mediating roles of loneliness and self-control separately (social exclusion was added as the *X* variable, smartphone addiction was added as the *Y* variable, loneliness as well as self-control were added as the mediator separately); this program (model 6) was used to examine the sequential mediating effect as well (social exclusion was added as the *X* variable, smartphone addiction was added as the *Y* variable, loneliness and self-control were added as the mediators sequentially; Hayes, 2017). The bootstrap approach was used to obtain the 95% bias-corrected confidence intervals (CI) of these mediating effects. This method would repeatedly sample from the original data set using random samples of size *n* (identical to the size of the sample in the original data set); next, the parameters (such as confidence intervals) would be evaluated by using each bootstrap sample; finally, the statistical significance could be identified (Jung et al., 2019). The confidence interval (CI) referred to the interval between a parameter estimate’s lower and upper limits at a certain confidence level (Jung et al., 2019). If a 95% CI included zero, the effect would be regarded as non-significant. Due to the fact that “bootstrapping provides the most powerful and reasonable method of obtaining confidence limits for specific indirect effects under most conditions” (Preacher and Hayes, 2008), and according to the recommendation of the previous study (Preacher and Hayes, 2008), the bias-corrected bootstrap estimates were performed based on 5,000 bootstrap samples.

## Results

### Preliminary analyses

The descriptive statistics and zero-correlations for all the study variables are displayed in Table 1. As expected, social exclusion was positively correlated with loneliness (*r* = 0.535, *p* < 0.01) and smartphone addiction (*r* = 0.328, *p* < 0.01), and negatively correlated with self-control (*r* = −0.385, *p* < 0.01). Besides, for the individuals with higher levels of loneliness (*r* = 0.339, *p* < 0.01) and low levels of self-control (*r* = −0.541, *p* < 0.01), they were more likely to be smartphone addicts. In addition, loneliness was negatively associated with self-control (*r* = −0.389, *p* < 0.01).

**Table 1:** Descriptive statistics and zero-order correlations of variables.

	Mean	SD	1	2	3	4
1. SE	17.347	5.997	1			
2. LON	11.189	3.602	0.535^**^	1		
3. SC	22.960	4.129	−0.385^**^	−0.389^**^	1	
4. SMA	32.691	10.333	0.328^**^	0.339^**^	−0.541^**^	1

SE, Social Exclusion; LON, Loneliness; SC, Self-Control; SMA, Smartphone Addiction.

** *p* < 0.01.

### Social exclusion as a predictor of smartphone addiction

A linear regression analysis was conducted to test the effect of social exclusion on smartphone addiction. Results indicated that social exclusion had a significant and positive impact on smartphone addiction (*b* = 0.565, *p* < 0.01). This result verified the first hypothesis (H1) of the present study.

### The mediating role of loneliness

PROCESS macro program (Model 4) was used to test the mediating effect of loneliness on the relationship between social exclusion and smartphone addiction. The results indicated that at step 1, social exclusion positively predicted loneliness (*b* = 0.322, *p* < 0.01); at step 2, social exclusion (*b* = 0.355, *p* < 0.01) and loneliness (*b* = 0.655, *p* < 0.01) positively predicted smartphone addiction. The direct effect of social exclusion on smartphone addiction was significant and positive (*b* = 0.355, 95% CI = [0.200, 0.510]); the indirect effect of social exclusion on smartphone addiction *via* loneliness was significant and positive (*b* = 0.211, 95% CI = [0.130, 0.312]). Therefore, loneliness partially mediated the association between social exclusion and smartphone addiction, the second hypothesis (H2) was supported.

### The mediating role of self-control

The mediating effect of self-control on the association between social exclusion and smartphone addiction was examined by adopting the PROCESS macro (model 4) as well. The results indicated that at step 1, social exclusion negatively predicted self-control (*b* = −0.265, *p* < 0.01); at step 2, social exclusion (*b* = 0.242, *p* < 0.01) and self-control (*b* = −1.220, *p* < 0.01) significantly predicted smartphone addiction. The direct effect of social exclusion on smartphone addiction was significant and positive (*b* = 0.242, 95% CI = [0.114, 0.369]); the indirect effect of social exclusion on smartphone addiction *via* self-control was significant and negative (*b* = 0.324, 95% CI = [0.251, 0.405]). Therefore, self-control partially mediated the association between social exclusion and smartphone addiction, the third hypothesis (H3) was confirmed.

### The sequential mediation model

The PROCESS macro (model 6) was applied to test the sequential mediating roles of loneliness and self-control in the relationship between social exclusion and smartphone addiction. The results are presented in Table 2. Social exclusion was significantly and positively related to loneliness (*b* = 0.322, *p* < 0.01) and negatively related to self-control (*b* = −0.171, *p* < 0.01). Besides, loneliness negatively predicted self-control (*b* = −0.293, *p* < 0.01), and positively predicted smartphone addiction (*b* = 0.314, *p* < 0.01). In addition, self-control was negatively correlated with smartphone addiction (*b* = −1.161, *p* < 0.01). Moreover, the direct effects of social exclusion on smartphone addiction was still significant (*b* = 0.156, *p* < 0.05) after controlling the effects of loneliness and self-control.

**Table 2:** Results of the sequential mediated model.

Effect	*b*	95%CI	
		Lower	Upper
Direct effect			
SE → LON	0.322^**^	0.280	0.363
SE → SC	−0.171^**^	−0.231	−0.111
LON → SC	−0.293^**^	−0.394	−0.193
SE → SMA	0.156^*^	0.014	0.299
LON → SMA	0.314^**^	0.079	0.552
SC → SMA	−1.161^**^	−1.351	−0.972
Indirect effect			
SE → LON → SMA	0.101	0.028	0.181
SE → SC → SMA	0.199	0.132	0.276
SE → LON → SC → SMA	0.110	0.071	0.155
Total Indirect Effect	0.410	0.316	0.516

SE, Social Exclusion; LON, Loneliness; SC, Self-Control; SMA, Smartphone Addiction.

* *p* < 0.05;

** *p* < 0.01.

As far as the indirect effects were considered, the pathway of “social exclusion → loneliness → smartphone” was significant (indirect effect = 0.101, 95% CI = [0.028, 0.181]). The indirect effect of social exclusion on smartphone addiction through self-control was significant (indirect effect = 0.199, 95% CI = [0.132, 0.276]). Besides, the indirect effect of social exclusion on smartphone addiction through loneliness and self-control in sequence was significant (indirect effect = 0.110, 95% CI = [0.071, 0.155]). Moreover, the total indirect effect was significant as well (indirect effect = 0.110, 95% CI = [0.316, 0.516]). These results indicated that loneliness and self-control partially and sequentially mediated the association between social exclusion and smartphone addiction, which supported the fourth (H4) hypothesis.

## Discussion

The relationship between social exclusion and smartphone addiction has not been widely studied, and little is known about the mediating mechanisms behind this association as well. According to the related theories and empirical studies, by adopting the process macro program, the present study established a sequential mediation model to test the relationship between the two variables and the mediating effects of loneliness and self-control on this association. The main findings and implications were listed and discussed as follows.

### The relationship between social exclusion and smartphone addiction

Previous studies have confirmed that social exclusion is one antecedent of Facebook addiction as well as internet addiction (Poon, 2018; Lim, 2019; Arslan and Coşkun, 2021). Some scholars have also found that social exclusion can positively and significantly predict substance use disorder and other addictive behaviors (Rabinovitz, 2014; Bacon and Engerman, 2018; Wesselmann and Parris, 2021). Likewise, consistent with H1, the results of the present study also demonstrated that social exclusion is a significant and positive predictor of smartphone addiction. According to the interpersonal model of addiction relapse (Leach and Kranzler, 2013), the aversion feeling associated with social pain is caused by the decreased endogenous opioid activity during and after the experience of social exclusion. When individuals were exposed to social exclusion, they appear to undergo “endogenous opioid withdrawal” which is marked by intense craving and significant negative emotion. Some early researchers also considered that addictive behavior may serve as an alternative for social bond (Panksepp et al., 1978). Besides, communicating with others is one of the most important functions of smartphones. This function can provide a virtual world for individuals who suffer from a lack of social connections. The “poor get richer” model indicated that the poor may use smartphones as compensatory tools to alleviate their social difficulties and increase their social networks (Amichai-Hamburger et al., 2008). As a result, people who have been socially excluded may be vulnerable to using smartphones to alleviate their aversion feelings. This will contribute to smartphone addiction.

### The mediating role of loneliness

Results of the current study indicated that H2 was confirmed: loneliness partially mediated the relationship between social exclusion and smartphone addiction. Specifically, social exclusion was positively correlated with loneliness, which in turn resulted in a higher level of smartphone addiction.

For the first path of the indirect effect, some evidence might be helpful for understanding the relationship between social exclusion and loneliness. As far as the concepts were considered, social exclusion refers to the experience of being physically or emotionally separated from others (Fung et al., 2016). In line with the emphasis on the experience of being separated from others in social exclusion, the sensation of being kept apart from other individuals has been colloquially termed loneliness (Weiss, 1973). From this point of view, it is reasonable to comprehend the positive association between the two variables. Besides, empirical studies have confirmed that loneliness is one of the negative consequences of social exclusion. According to the information theory of emotion (Simonov, 2013), negative emotions originate from the difference between available and indispensable information. Consistent with this theory, previous research has indicated that loneliness results from the difference between the desired and achieved interpersonal interactions (Cacioppo et al., 2014). Due to the fact that social exclusion impedes individuals from achieving desired relationships, consequently, the deficiency of social acceptance, inclusion, and support will contribute to the occurrence and development of loneliness. In addition, the need-to-belong theory and the temporal need-threat model (Williams, 2009; Baumeister, 2011) also posited that social exclusion may threaten individuals’ fundamental needs – belonging, which will result in loneliness feelings. Based on empirical and theoretical evidence, the relationship between social exclusion and loneliness could be comprehended.

For the second path of the indirect effect, loneliness was positively associated with smartphone addiction. This finding was consistent with the previous studies which demonstrated that loneliness is a risk factor for smartphone addiction (Enez Darcin et al., 2016; Mahapatra, 2019; Kayis et al., 2021). According to the compensatory internet-use model, the purpose that people go online is to alleviate negative affects or evade real-life events (Kardefelt-Winther, 2014). Therefore, loneliness, whether it serves as one of the undesirable experiences or a negative life issue, may lead people to be addicted to smartphones. The uses and gratifications theory posited that individuals’ smartphone use was motivated by their underlying psychological requirements (Katz, 1974). Because loneliness is characterized by the absence of belonging (Franklin and Tranter, 2021), and belonging needs have been regarded as one fundamental human motivation (Baumeister and Leary, 1995), consequently, lonely individuals were more likely to use smartphones excessively, intensively and endlessly. This would result in smartphone addiction.

In summary, the current study provided evidence that social exclusion might threaten individuals’ belonging needs, which would lead to loneliness. Subsequently, lonely people would select smartphones to alleviate their negative experiences as well as meet their basic psychological requirements for interaction with others and inclusion by others.

### The mediating role of self-control

In line with H3, the results of the current study indicated that self-control partially mediated the link between social exclusion and smartphone addiction. Specifically, social exclusion experiences resulted in lower levels of self-control, which in turn brought about a higher severity of smartphone addiction.

For the first path of this indirect effect, the results of this study were consistent with the prior studies. Some scholars have indicated that social exclusion is not only a direct predictor of self-control (Crescioni and Baumeister, 2009; Burson et al., 2012; Xiaojun et al., 2017), but can also contribute to negative affects and experiences such as loneliness, depression, and anger (Fung et al., 2016; Feng et al., 2019; Carlyle et al., 2020; Arslan, 2021), which will indirectly decrease self-control (Chester et al., 2016). This might be because social exclusion threatens individuals’ basic psychological requirements (Williams, 2009; Baumeister, 2011), which will motivate people to do something actively to compensate for their psychological distress or find some substitutes to console themselves. The greater the extent of social exclusion they perceived, or the more social exclusion situations they experienced, the greater the motivation to fulfill their needs would be, and the lower the level of self-control they could possess. Besides, according to the strength model of self-control (Baumeister et al., 2007), managing emotions and overcoming unwanted motivations will deplete self-control resources. Because social exclusion might cause negative feelings and threaten people’s basic psychological needs, individuals’ self-control would decrease after being socially excluded. Therefore, self-control as one of the detrimental consequences of social exclusion could be understood.

For the second path of this indirect effect, self-control was negatively associated with smartphone addiction. This finding was consistent with the previous studies which demonstrated that low self-control is a risk factor for smartphone addiction (Kim et al., 2016, 2018; Sok et al., 2019). Following what had been discussed in the prior paragraph, when people’s self-control resources were depleted, they would not be able to control or regulate their behaviors and suppress their undesirable actions; besides, they might fail to set and keep long-term goals (Sok et al., 2019). Empirical studies have also found that the consumption of self-control strength can promote approach inclinations and reward-seeking actions (Giacomantonio et al., 2014). Due to the fact that smartphones have multiple functions that could be used for numerous activities, individuals low in self-control are vulnerable to being attracted to smartphones and tend to use smartphones immoderately. This might lead them to be addicted to this intelligent device. Moreover, a prior study has confirmed that individuals with low self-control are more likely to be socially excluded (Walters, 2016). Consequently, the impact of social exclusion would exacerbate people’s self-control and subsequently contribute to smartphone addiction.

In summary, the current study provided evidence that social exclusion might threaten individuals’ psychological needs and bring about negative experiences, which not only motivated them to find compensation to fulfill their requirements but also depleted their self-control resources. Subsequently, people low in self-control would use smartphones to compensate for their psychological requirements and alleviate their negative feelings, which would contribute to smartphone addiction.

### The sequential mediating effects of loneliness and self-control

The results of the present study supported H4: loneliness and self-control sequentially mediated the link between social exclusion and smartphone addiction. Specifically, socially excluded individuals were more likely to experience loneliness, which in turn depleted their self-control strength and eventually brought about a higher severity of smartphone addiction. According to the I-PACE model (Brand et al., 2016), social exclusion, which acts as one type of social cognitions, is one of the core characteristics of the individuals; the negative affects and reduced executive functioning represent the mediators. The interaction of these indicators can contribute to smartphone addiction. Likewise, according to Social Cognitive Theory (SCT), “environmental influences, cognitive and personal factors, and behaviors all operate as interlocking determinants that affect each other bidirectionally” (Bandura, 1985). In the present study, social exclusion, loneliness, and self-control were regarded as the environmental and personal factors that gave rise to the occurrence and development of the behavioral consequence -- smartphone addiction. Therefore, the I-PACE model and SCT offered holistic perspectives to explain the relationships between these variables, and in turn, the results of the current study also provided empirical evidence for the two theories.

## Conclusion

The present study examined the association between social exclusion and smartphone addiction as well as the mediating effects of loneliness and self-control on this relationship. Results indicated that: (1) social exclusion was a positive predictor of smartphone addiction; (2) loneliness and self-control separately mediated the association between social exclusion and smartphone addiction; and (3) loneliness and self-control sequentially mediated the relation between social exclusion and smartphone addiction. These results were conducive to comprehending the relationship between the two study variables as well as the underlying mechanism of this relationship. Some suggestions for future studies were listed as follows: First of all, because the present study was a cross-sectional design, the longitudinal design was encouraged to investigate the stable influence and the mediating effects of loneliness and self-control of social exclusion on smartphone addiction. In addition, social exclusion could lead to other detrimental influences on individuals’ mental health, such as low self-esteem and a need to belong (Williams, 2009). Since these mental distress might also contribute to the development of smartphone addiction, future research was required to investigate different mediating mechanisms of the association between social exclusion and smartphone addiction. Moreover, future studies were recommended to explore effective approaches for reducing social exclusion, which would contribute to reducing the risk of smartphone addiction.

## Data availability statement

The original contributions presented in the study are included in the article/Supplementary material, further inquiries can be directed to the corresponding author.

## Ethics statement

The studies involving human participants were reviewed and approved by the College of Psychology Inner Mongolia Normal University. Written informed consent for participation was not required for this study in accordance with the national legislation and the institutional requirements.

## Author contributions

XZ and HB: funding acquisition. XY, XZ, and BL: investigation. HY: writing – original draft. HB: writing - review and editing. All authors contributed to the article and approved the submitted version.

## Funding

This study was supported by Inner Mongolia Natural Science Foundation, Exercise improves executive function in patients with chronic pain (2021MS03099), Social Science Planning Project in Inner Mongolia, Evaluation System and Intervention Research of Adolescent Health Literacy in Inner Mongolia under Healthy China Strategy (2021NDB130), and Baotou Medical College’s research project of “Wen Xue,” “Wei Xue” and “Jian Xue,” a study on emotional adjustment strategies and influencing factors of college students under the influence of public emergencies (2021BYWWJ-YB-37).

## Conflict of interest

The authors declare that the research was conducted in the absence of any commercial or financial relationships that could be construed as a potential conflict of interest.

## Publisher’s note

All claims expressed in this article are solely those of the authors and do not necessarily represent those of their affiliated organizations, or those of the publisher, the editors and the reviewers. Any product that may be evaluated in this article, or claim that may be made by its manufacturer, is not guaranteed or endorsed by the publisher.
